# Misuse of "study drugs:" prevalence, consequences, and implications for policy

**DOI:** 10.1186/1747-597X-1-15

**Published:** 2006-06-09

**Authors:** Steve Sussman, Mary Ann Pentz, Donna Spruijt-Metz, Toby Miller

**Affiliations:** 1 Preventive Medicine and Psychology, Institute for Health Promotion and Disease Prevention Research, University of Southern California, 1000 South Fremont, Unit #8, Alhambra, CA 91803, USA; 2 Preventive Medicine, Institute for Health Promotion and Disease Prevention Research, University of Southern California, 1000 South Fremont, Unit #8, Alhambra, CA 91803, USA; 3 Sociology, University of California-Riverside, 1206 Watkins Hall, Riverside, CA 92521, USA

## Abstract

**Background:**

Non-medical/illegal use of prescription stimulants popularly have been referred to as "study drugs". This paper discusses the current prevalence and consequences of misuse of these drugs and implications of this information for drug policy.

**Results:**

Study drugs are being misused annually by approximately 4% of older teens and emerging adults. Yet, there are numerous consequences of misuse of prescription stimulants including addiction, negative reactions to high dosages, and medical complications. Policy implications include continuing to limit access to study drugs, finding more safe prescription drug alternatives, interdiction, and public education.

**Conclusion:**

Much more work is needed on prescription stimulant misuse assessment, identifying the extent of the social and economic costs of misuse, monitoring and reducing access, and developing prevention and cessation education efforts.

## 

Prescription stimulants that have been used non-medically/illegally often have been referred to as "study drugs" or "cramming drugs," and sometimes "kiddy coke." Methylphenidate (e.g., Ritalin) is the most widely misused and researched of these drugs and has been referred to as "Vitamin R", "Skippy", "the Smart Drug", "Smarties", "Poor Man's Cocaine", "West Coast", and "R Ball" [1-4]. While prescribed for sufferers of Attention-Deficit/Hyperactivity Disorder (ADHD), and costing approximately $0.50 a pill when obtained licitly, prescription stimulant pills can cost $3 to $15 each when sold illicitly [1-3,5]. Several recent news articles have been published about potential dangers of misuse of study drugs, and in February 2006, a Food and Drug Administration advisory panel urged that the strongest possible safety warning (the "black box" warning for cardiac problems and sudden death in pediatric patients) be used on these drugs [6,7]. This review will describe briefly the history of prescription stimulants, their prevalence of misuse among emerging adults [8], and potential negative consequences. Policy implications also will be suggested.

## Brief history and current status of study drugs

Methylphenidate was first created in 1944 as part of a search for a non-addictive stimulant, and it was suggested as a means of regulating children's behavior in 1963, to control "hyperkinesis" [9]. Eventually such problematic behavior among children became labeled as "Attention-Deficit/Hyperactivity Disorder" (ADHD; [10]). ADHD refers to a constellation of dysfunctions that hinder attention regulation, motor behavior, impulsivity, emotional expression, and planning [11].

By 1970, 15 different pharmaceutical companies manufactured over 30 kinds of prescription stimulant-type products. Eventually a very large ADHD drug market developed, which has been dominated by stimulants. These currently include methylphenidate (brand names: Ritalin, Ritalin SR, Methylin, Methylin ER, Metadate, Metadate ER, Concerta), demethylphenidate (brand name: Focalin) and amphetamine preparations including D-amphetamine (brand names: Dexedrine, Spansule, Dextrostat), methamphetamine (brand name: Desoxyn) and D, L-amphetamine (brand names: Adderall, Adderall XL). People have observed the misuse of study drugs among those not diagnosed with ADHD, particularly among emerging adults [12]. "Emerging adulthood" is a recently "coined" developmental period, referring to young people 18–25 years of age, who bridge the gap between adolescence and young adulthood [8]. Misuse has been observed particularly in college settings [12,13]. Many misuse these drugs to help keep alert and concentrate as they prepare ("cram") for tests or complete term papers (though, of course, people may misuse them for a variety of reasons [12,13])--hence the term "study drugs" [14].

## Prevalence of non-medical use of prescription stimulants: college settings

Regarding college prevalence data and policy, the most frequently studied prescription stimulant is methylphenidate. From 1990 to 2000, use of methylphenidate increased five-fold in the United States, which consumes approximately 90% of all methylphenidate [12,15]. The Monitoring the Future research group has been studying the annual prevalence of use of methylphenidate (measured as "Ritalin," as a stand-alone question) among teens and emerging adults since 2001 [[16]; also see [17]]. The annual prevalence among 8^th^, 10^th^, and 12^th ^graders has averaged 2.7%, 4.3%, and 4.5%, respectively, over the period from 2001 to 2004. Its use among college students has averaged 5.0% over this period, whereas its use among non-college emerging adults has averaged approximately 2.9% over this period. (About 25% of these users use methylphenidate about once a month [16,17].)

Its use remains at approximately 3.5% through 24 years age. At 25 years of age its use decreases to 1.5% and then below 1% at 29–30 years of age [16]. Thus, use of this study drug appears to peak from the ages of 16 to 24 years of age (older adolescence through emerging adulthood), following the same course of use prevalence as other drugs such as alcohol and illicit drugs [also see [18]]. Males are relatively likely to use methylphenidate (3.7% versus 1.6%), and use appears slightly higher in the southern states in the Monitoring the Future survey (2.8% versus 2.0–2.5%), and in very large urban areas (about 3.0% versus 1.9–2.7%). Methylphenidate appears most likely to be used by male college students in large cities [16].

Several studies have been completed to discern prevalence and reasons for non-medical use of prescription stimulants. Simoni-Wastila & Strickler [19] examined 1991–1993 data from the National Household survey on Drug Abuse (NHSDA), a nationally representative, randomized-block selected household sample of 12-year olds and older. They found that 1% of the U.S. population had used prescription stimulants non-medically in the past year, and a fifth of them reported problem use (e.g., inability to cut down use), though they failed to find significant demographic, health status, or drug use correlates of illicit problem stimulant use. Certainly, the age range of highest lifetime use of prescription stimulants is from 18–25 [20], approximately 5%, and at least doubling percentage figures among other age categories viewed across multiple years of this survey.

In a more recent study, McCabe and colleagues [12] examined the prevalence rates and correlates of non-medical use of prescription stimulants (methylphenidate, D-amphetamine, or D,L-amphetamine) among U.S. college students. One hundred and nineteen nationally representative 4-year colleges in the United States were selected and a sample of 10,904 randomly selected college students in 2001 were examined via self-report surveys. The life-time prevalence of non-medical prescription stimulant use was 6.9%, past year prevalence was 4.1% (but ranged from 0% to 25% across colleges), and past month prevalence was 2.1%. A total of 5.8% of males and 2.9% of females reported annual use of non-prescribed stimulants. In addition, 2.8% of males (1.6% of females) reported use in the past month. Whites were relatively likely to misuse prescription stimulants compared to African American, Asian, or other groups (annual use: 4.9% versus 1.6%, 1.3%, and 3.1%, respectively). The prevalence of non-medical use of prescription stimulants among students attending historically African American colleges and universities was low [12].

Past year rates of non-medical use ranged from zero to 25% at individual colleges. Non-medical use was higher among college students who were members of fraternities and sororities (annual use: 13.3% if living in a fraternity or sorority house, 3.5%-4.5% otherwise; 8.0% versus 1.8%–2.5% last month use), and earned lower grade point averages (annual use: B or lower average, 5.2%; B+ or higher, 3.3%). Rates were higher at colleges located in the north-eastern region of the U.S. (6.3% annual use), and southern region (4.6%), then other regions (2.8–3.2%), and colleges with more competitive admission standards (5.9% versus 1.3–4.5% annual use). Non-medical prescription stimulant users were more likely to report use of alcohol, cigarettes, marijuana, ecstasy, and cocaine, raising the possibility that use of prescription stimulants for non-medical purposes may be related as much to an addiction disorder as to an aid to study.

These studies examined a variety of prescription stimulant drugs, not just methylphenidate. Thus, arguably, a 4.1–5.4% annual prevalence represents the most accurate statement regarding those at highest prevalence of non-prescription use.

Several single-university sample surveys of college students have reported a range of use reports. Massachusetts, Michigan, New Hampshire, Maine, Florida, Pennsylvania, Wisconsin, and Texas samples are reported herein. In one random sample at a Massachusetts college of liberal arts (n = 283), 16.6% of the sample reported having taken methylphenidate for fun (non-medical purposes), and 12.7% reported having snorted methylphenidate; a majority of the self-reported users were under 24 years of age [21].

Teter, McCabe and colleagues [22] administered a survey to 2250 randomly selected undergraduates at the University of Michigan (in 2001) and found a 3% annual prevalence of illicit methylphenidate use, which was positively associated with other licit and illicit drug use (but not with gender or ethnicity). The same research group [23] assessed the prevalence and motives for illicit use of prescription stimulants (methylphenidate [two brands], D-amphetamine, and D, L-amphetamine) to a random sample of 9,161 undergraduate college students at the University of Michigan (surveyed in 2003). Of the study participants, 8.1% reported lifetime and 5.4% reported past-year illicit use of prescription stimulants. The most prevalent motives given for illicit use of prescription stimulants were to (1) help with concentration (58% of lifetime illicit users), (2) increase alertness (43%), and (3) provide a high (43%). Eighty-six percent reported not being prescribed these drugs in their lifetime. Men were more likely than women to report illicit use of prescription stimulants (9.3% versus 7.2%), and Whites and Hispanics (9.5% and 8.9%, respectively) were more likely to use them than African American or Asian students (2.7% and 4.9%, respectively). Illicit use of prescription stimulants was associated with elevated rates of alcohol and other drug use, and total number of motives endorsed and alcohol and other drug use were positively associated.

These same authors [13] reported an additional study of prescription drug misuse and diversion (i.e., diverting use from those prescribed the medication to those not prescribed the medication), in the same cohort as the Teter et al. [23] study. The annual illicit use of stimulant medication was 5%, and the illicit use-medical use ratio for stimulant medication (overall = 2.45) was the highest among the four classes of prescription drugs examined (overall ratios for the other three drug types, sleeping, sedative/anxiety, and pain medications were less than 1.0). Medical users of stimulants for attention deficit hyperactivity disorder were the most likely to be approached to divert their medication (54% of them reported being approached to sell, trade, or give away their medication). Illicit users of medical stimulants were relatively likely to use other drugs (odds ratios varied from 6.00 for binge drinking to 21.25 for annual cocaine use).

White, Becker-Blease, & Grace-Bishop [5] examined illicit use of stimulant medication among undergraduate and graduate students at the University of New Hampshire. Of 1,025 randomly sampled participants, 16% reported misusing stimulant medication (96% of those that specified a medication preferred to misuse methylphenidate, 2% misused D, L-amphetamine). Ninety-percent of these subjects reported never receiving an ADHD diagnosis. Results failed to differ as a function of gender. Most used pill form (55%), but 40% had used intranasally. Of those that misused prescription stimulants, about 50% reported misuse 2–3 per year, whereas the other 50% reported misuse at least once per month. Reasons for misusing prescription stimulant medication (i.e., illegal use) included improving attention, partying, reducing hyperactivity, and improving grades.

In a convenience sample survey of 150 undergraduates at a small U.S. college (in Maine), 35.5% took prescription amphetamines (D,L-amphetamine, methylphenidate, or D-amphetamine) illegally. A total of 24% of the illicit users used amphetamines to study, but 19.3% used them in combination with alcohol for recreational reasons [24].

There have been several unpublished surveys conducted by individual colleges. For example, Kapner [1] summarized four unpublished surveys of methylphenidate use from college reports, and found that 1.5% of students surveyed at the University of Florida in 2002 reported using methylphenidate recreationally in the last 30 days, 9% of those undergraduates surveyed at the University of Pennsylvania in 2000 had used someone else's ADHD prescription medication, 20% of those students surveyed at the University of Wisconsin, Madison, in 1998, had illegally taken an ADHD medication at least once, and 2% and 1.5% of those students surveyed at the University of Texas in 1997 had used methylphenidate illegally in their lifetimes and the past year, respectively. The sampling details of these reports were not disclosed and could not be located; thus, these results must be taken with caution. In the studies involving college students, college student participation rates among those "randomly" selected varied from 20–60%. Thus, replication studies with recruitment of a greater percentage of the targeted sample still are needed, if this can be accomplished in college settings.

To summarize, lifetime, past year, and past month illicit use of prescription stimulants among emerging adults appears to vary widely while averaging approximately 7%, 4%, and 2%, nationally. Use is most prevalent among white or Hispanic male college students, who are associated with fraternities, struggling with their grades, and who generally live in larger urban areas (though not always) in northeastern or southern regions of the U.S. These youth also tend to use other drugs particularly cannabis, alcohol, MDMA, and cocaine [also see [25]]. They use study drugs to enhance their study and social life, and sometimes to stay awake while using another drug such as alcohol.

### Routes of Administration

Prescription stimulants have been misused through oral, intranasal, and intravenous routes of administration. Oral misuse of prescription stimulants results in noticeable symptoms if taken in high doses. Encapsulated extended-release formulas can be misused and abused as well as the older shorter-release formulas by breaking open a capsule and snorting the contents. A series of case studies have indicated intranasal and injection abuse of study drugs [4,26,27]. In one study of New Hampshire undergraduate and graduate students [5], the preferred method of use among study drug misusers was oral (55%), intranasal (40%), and "Other" (4%). The route of administration of the "other" category was not specified, but it is possible that intravenous use was being referred to. More disconcerting, 79% of illicit users were not at all concerned about using these study drugs.

## Potential negative consequences of "study drug" use

There are three general potential negative consequences of misusing study drugs. These include: (a) potential for addiction, (b) potential for reactions to high doses, (c) and potential for medical complications. These three consequences are discussed next.

### Addiction

A review of 60 studies suggested that the reinforcing or subjective effects of methylphenidate (in 80% of these studies) functions similarly to d-amphetamine or cocaine (i.e., as a reinforcer, in drug discrimination substitution, and subjective effects such as producing a "high" or "rush"), and that there is definite abuse potential [4]. Tolerance develops and characteristic stimulant withdrawal symptoms have been reported including fatigue or exhaustion, depression, unpleasant and vivid dreams, insomnia or hypersomnia, increased appetite, psychomotor retardation or agitation, or irritability [2,11,26-28]. Similar effects may be expected with all prescription stimulants.

Stimulants tend to increase or augment dopaminergic (reward, anticipation) and serotoninergic (self-administration initiation, maintenance of pleasure) neurotransmission [29]. Methylphenidate appears to work by blocking pre-synaptic dopaminergic transporters [29], and does not appear to affect the serotonergic system [30]. Its effects on dopaminergic transmission are similar to cocaine, and may lead to similar consequences through intranasal administration or injection. For example, in 23 case studies of intravenous methylphenidate use, the pattern of withdrawal and toxicity symptoms were similar to that of cocaine and amphetamine [26,27].

Oral use produces its effect in approximately an hour compared to a couple of minutes for other routes of methylphenidate (or cocaine) administration. Oral intake does not produce nearly as much reinforcing effect and, hence, has much less abuse potential [31], depending on the dose taken. Still, one should not stop using oral methylphenidate abruptly if one has been using it consistently, because it will produce withdrawal symptoms characteristic of other stimulants [2,11,18]. Much more work is needed on the study of how routes of administration may interface with misuse and addiction to prescription stimulants.

### High doses

At high doses, study drugs can produce symptoms such as emotional lability, anxiety, twitchiness, aggressiveness, loss of appetite, confusion, dizziness or blurred vision, insomnia, headaches, sweating, and dryness of the mouth and eyes [5,11,26]. Methylphenidate and other study drug misuse may result in formification hallucinations (e.g., one may have a tactile perception as if there are bugs under ones skin), repetitive behaviors ("tweaking"), and bizarre delusions (e.g., personalization of objects, paranoia) [1,11,21], if used chronically at high doses, especially by intranasal administration or injection.

In 1990, there were about 271 emergency room reports involving methylphenidate, 1,727 in 1998, and 1,478 in 2001 [32]. The total number of emergency department visits resulting from use of all psychotherapeutic CNS stimulants was 4091 in 1998, 3644 in 1999, 3336, in 2000, 3146 in 2001 and 3275 in 2002 [33]. There are approximately 25 emergency room deaths per year among up to 3 million users of prescription stimulant drugs (including both those medically prescribed and not prescribed these drugs). Thus, the likelihood of dying from such drugs appears to be approximately 1 in 120,000 [32].

### Medical complications

Most study drugs raise blood pressure and may place users at risk for heart attacks and stroke [5,6]. For example, side effects may include irregular heartbeat and very high blood pressure [1,15]. Thus, use is contraindicated if one has a history of high blood pressure or other cardiovascular-related concerns. In addition, use is contraindicated among those suffering from psychiatric conditions including severe anxiety, glaucoma, motor tics (or Tourette's syndrome), psychotic conditions, depression, a seizure disorder, or a history of drug abuse. Also, one should not use prescription stimulants if one has experienced a narrowing of ones gastrointestinal tract or a damaged liver. Finally, use also is contraindicated if one is taking other prescribed drugs particularly monoamine oxidase (MAO) inhibitors [e.g., see [2,34]].

Intravenous use of prescription stimulants is particularly dangerous. In particular, intravenous (IV) abuse of methylphenidate may result in talcosis. Talcosis is a reaction to talc, a filler and lubricant in methylphenidate and other oral medication. This inflammation reaction occurs in the lungs and related consequences include lower lobe panacinar emphysema [35].

## Implications for policy change

Current rates of study drug misuse show the potential to increase dramatically in direct proportion to the prevalence of manufacture, prescription, and general availability [36]. Five types of policy change are recommended to curb the misuse of these drugs.

First, limiting access to prescription stimulants may be a very important approach [36]. Those who are correctly prescribed ADHD medications could be involved in a monitoring system to try to make sure that they are not serving as suppliers to others [12,23]. Fortunately, 21 states in the U.S. use some sort of prescription monitoring program (PMP) to monitor the use of abuse-able prescription drugs [37]. All these states include Schedule II stimulants as one of the drug categories being monitored and, often, lower scheduled ones as well.

Second, facilitating development of more safe alternative types of drugs may be very important as well [36]. There is a relatively novel drug, Lilly's atomoxetine (brand name: Strattera), which has the advantage of not being classed as a central nervous system stimulant. Atomoxetine is a norepinephrine transporter inhibitor. It has similar side effects as the other drugs as well as potentially leading to urinary hesitation or retention. However, it probably has less abuse potential [11]. There also exist novel pharmaceutical delivery systems that have been shown to be less prone to abuse (e.g., the Concerta formulation of methylphenidate [13]). Possibly, use of these types of prescription drugs by those suffering from ADHD may lead to decreased prevalence of prescription drug misuse through diversion to those not suffering from ADHD. A recent study indicates very little misuse of the extended-release formula of methylphenidate in 2002 [18].

The third change pertains to policies aimed at interdiction. These policies would be enforced by local law enforcement personnel to disrupt unauthorized points-of-sale or distribution. However, according to an article by Pentz and colleagues based on a review of interdiction policies aimed at other drugs, this type of policy has appeared to be largely ineffective [36].

The fourth is policies aimed at warning the public as well as users about the negative consequences of illicit use of prescription stimulants (study drugs). The FDA while having declared most of these drugs as controlled substances, should provide a wider array and more visible platforms (e.g., on labels, public announcements) discouraging misuse and highlighting negative consequences of use, unauthorized sales, and distribution of these drugs.

The fifth, and perhaps the most promising, are policies aimed at institutionalization of education about study drugs. Education could include changes in physician treatment regimen protocols that are formalized by the AMA, including requirements for additional continuing education for pediatricians and family physicians to learn about non-medical treatment options and the potential for diversion of these medications. Potential for diversion also should be instructed to medication users. As mentioned earlier in this review, at least one study has examined the prevalence rates of prescribed college stimulant users being approached to divert their stimulant medication [13]. Of the undergraduate students who were medically prescribed stimulant medication for ADHD, approximately 54% had been approached to divert their medication (e.g. sell, trade or give away) in the past year. Means to protect these students from those that might lure or coerce them into providing their stimulant medication to others are needed. Possibly, college-level prevention programs could be developed to include resistance to offers of study drugs other than those prescribed by a physician. One recommendation that has been made in the New York University Health Center is that those persons prescribed ADHD drugs while in college should keep their drug in a private location, and give reasons for not providing the drugs to others (e.g., to avoid a potential allergic reaction, not enough to share, claim that one has stopped using the drug; [38])

Instruction in good study skills is one way to try to bypass reliance on cramming for exams and associated prescription medication diversion. There are a variety of self-help courses that universities provide to help improve study skills (e.g., at Virginia Polytechnic Institute [39]). These skills involve time management (e.g., scheduling classes and study times, keeping track of tasks to be completed [task lists, charting work tasks on a timeline]), placing a priority on studying (treating it as a full-time job), identifying and removing time wasters, and learning how to concentrate better (e.g., removing distractions from environment, studying in fixed locations, using a timer to increase concentration time, taking scheduled breaks). Cognitive-behavior therapy is popularly suggested for treatment of study drug abuse, but little empirical data exists specifically on its use with these drugs [e.g., [26]].

## Conclusion

"Study drug" misuse deserves more study. It appears to be concentrated in certain groups for whom programming might be tailored (e.g., males who abide by a fraternity college lifestyle, persons with friends or associates who are prescribed the medication). Abuse occurs when used in rather high doses (orally) or when administered intransally or injected. Monitoring its spread among at risk populations is important. Aggressive marketing of study habit courses is needed. Youth that illegally use these prescription stimulant drugs are relatively likely to use other drugs and suffer from drug abuse [23,25]. They are also relatively likely to incur serious damage from improper administration of these drugs. Strong warning labels may help, but may be limited as a means of drug control [25,36]. Much more work is needed on prescription stimulant misuse assessment, reducing access, prevention, cessation, and on identifying the social and economic costs of its misuse.

## Abbreviations

None.

## Competing interests

The author(s) declare that they have no competing interests.

## Authors' contributions

SS did the reference search and the majority of the writing and editing.

MAP contributed most of the policy material and editing.

D A-M contributed some of the material on prescription stimulant consequences and editing.

TM, as an experienced writer on Ritalin abuse, provided a mentor role, contributed to material throughout, and editing.
